# Evaluation of ultrasound-guided fine needle aspiration biopsy (USG-FNAB) of thyroid nodules: 12 years of accepted diagnostic algorithm in Holycross Cancer Centre (HCC) in Kielce

**DOI:** 10.1186/1756-6614-6-S2-A58

**Published:** 2013-04-05

**Authors:** Jacek Sygut, Aldona Kowalska, Janusz Słuszniak, Jacek Heciak, Janusz Kopczyński, Dominik Sygut, Kornelia Niemyska, Stanisław Góźdź

**Affiliations:** 1 Pathology Department, Holycross Cancer Centre in Kielce, Kielce, Poland; 2 Endocrinology Department, Holycross Cancer Centre in Kielce, Kielce, Poland; 3 Oncological Surgery Department, Holycross Cancer Centre in Kielce, Kielce, Poland; 4 Radiology and Imaging Diagnostics Department, Holycross Cancer Centre in Kielce, Kielce, Poland; 5 Pathology Department, Medical University of Lodz, Lodz, Poland; 6 Holycross Cancer Centre Board, Kielce, Poland

## Introduction

Role and place of USG-FNAB in diagnosis of thyroid lesions were confirmed in recently published algorithm proposed by the recommendations of Polish Group of Endocrine Tumours.

## Aim of the study

Presentation of parameters describing thyroid USG-FNAB in HCC in Kielce.

## Material

Since year 2000 all FNABs, including thyroid gland, were performed and evaluated according to the accepted diagnostic algorithm. Between 2001 and 2012 FNAB was performed for 26361 patients, in 28079 biopsy sessions with 40815 ultrasonographically selected and biopsied thyroid lesions. Since year 2001, all data about subsequently surgically treated patients along with pathology reports and with previous FNAB data were currently gathered. Each correlated case gained true positive (TP), true negative (TN), false positive (FP) or false negative (FN) status. There were overall 1453 cases of thyroid malignancies of all 10142 correlated FNABs *vs.* subsequent surgical specimens in CORRELATION DATABASE between 2001-2012. In that period of time there were 343 TP, 1026 TN, 3 FP and 74 FN correlated cases of thyroid malignancies. On the basis of these data, sensitivity, specificity and overall accuracy for thyroid USG-FNAB were calculated.

## Methods

On the basis of annual data for true and false cases, overall sensitivity (OS), overall specificity (OSP) and overall accuracy (OA) were estimated. The trend for those values was evaluated.

## Results

For whole analyzed period of time (2001-2012) FNAB’s OS was 0.85, ranging from 0.68 to 1.0 and OSP was 1.0, ranging from 0.97 to 1.0. OA was 0.95, ranging from 0.9 to 1.0. In graphical analysis there was a strong gradual trend of increase in sensitivity since year 2007, with maintaining specificity approaching 1.0. Statistically significant strong positive trend of sensitivity value (R=0.76, p=0.004) was shown for each year, separately from the flow of time, in analyzed period of time.

## Conclusions

Applied in our department USG-FNAB method was characterized by statistically significant and regular increase of correct malignant tumour diagnoses (sensitivity) and overall accuracy with stable high level of correct benign lesions diagnoses (specificity).

